# Gingival crevicular blood: A diagnostic tool for diabetes mellitus

**DOI:** 10.6026/973206300210962

**Published:** 2025-05-31

**Authors:** Rahul K Chaudhari, Hitesh M Desarda, Keerthi Prasanna Chennuri, Aksharkumar J Patel, Neelima Chowdary Cherukumalli Kapalavayi, Praveen Kumar Gonuguntla Kamma

**Affiliations:** 1 Department of Oral and Maxillofacial Surgery, Government Dental College and Hospital, Jalgaon, Maharashtra, India; 2 Department of Periodontology and Implantology, Government Dental College and Hospital, Jalgaon, Maharashtra, India; 3 Department of Dental, University of Louisville Dental School, Kentucky, United states of America; 4 Department of Dental, Scott Family Dental Office, Thunder Bay, Ontario, Canada, North America; 5 Department of Dentist, Brident Dental and Orthodontics, Texas, United States of America; 6 Department of Dentist, Celebrate Dental and Braces, Texas, United States of America

**Keywords:** Gingival crevicular blood, diabetes mellitus, periodontitis, capillary finger prick blood

## Abstract

Patients with periodontitis are significantly more prone for undetected diabetes mellitus (DM). Gingival crevicular blood (GCB), which oozes during periodontal examination, can be utilized for screening DM patients. Therefore, it is of interest to compare the efficacy of GCB with capillary finger prick blood (CFB). A strong positive correlation was observed in CFB and GCB with 'r' values of 0.93 suggestive of highly statistically significant (P<0.0001).

## Background:

Diabetes mellitus (DM) is one of the major global epidemic diseases, significantly associated with mortality and morbidity, conferring a substantial burden on the health care system [1]. DM is characterized by high glucose levels associated with dysregulation of carbohydrate, fat and lipid metabolism [1, 2]. Factors responsible for increasing cases of DM are: altered food habits, stress and obesity. The prevalence of DM for all age groups worldwide was estimated to be 2.8% in 2000 and 4.4% in 2030 [2]. In 2021, the American Diabetes Association (ADA) updated diagnostic guidelines for DM, helps in differentiating between pre-diabetes and diabetes patients [3]. The traditional techniques used for the detection of DM are to venous blood or glycated hemoglobin level (HbA1c) [4]. These techniques take time and require sophisticated equipment. Self-monitoring devices (glucometers) have made it possible for diabetic's patients to have more control over their condition [4]. Glucometers measure blood glucose levels in few seconds using a small drop of capillary blood from a finger prick. Around half of total diabetic patients remain undiagnosed, as DM is asymptomatic in its early stage and can remain undiagnosed for many years. Early diagnosis may prevent long term complications [5]. Periodontitis has been proposed as the sixth complication of diabetes [6]. There is a proven two-way association between periodontal disease and DM [7-8]. The dentist may play an important role by participating in the diagnosis asymptomatic DM [9]. Bleeding from the gingival sulcus during routine periodontal examination, which is a mixture of capillary blood and gingival crevicular fluid, can be used for painless monitoring of blood glucose levels with glucometers [9]. Therefore, it is of interest to compare the blood glucose levels between gingival crevicular blood (GCB) and capillary finger prick (CFB) blood using a self-monitoring device (SMD).

## Materials and Methods:

The study participants were recruited from outpatient Department of government dental college and hospital, Jalgaon, Maharashtra, India. Total 200 patients (age range, 25 to 70 years) with gingivitis or periodontitis with positive bleeding on probing (BOP) were chosen for the present study. Exclusion criteria includes: bleeding disorder, antibiotic prophylaxis, severe systemic disease such as cardiovascular, renal, hepatic, immunologic disorders. Written informed consent was obtained from each patient. The study was reviewed and approved by the institutional ethical committee. Initially routine oral examination was done and following data was recorded: Probing depth (PD), Bleeding on probing (BOP), gingival bleeding index (GBI) and periodontal disease index (PDI). All this data was collected by single examiner. For routine periodontal examination Williams probe was used. One site with profuse Bleeding on Probing was chosen for testing blood glucose level. For testing of the GCB sample, readily available SMD (OneTouch Horizon Blood Glucose Monitoring System) with a compact design was selected. The glucometer was placed intraoral with the test strip in place and blood was allowed to flow on its reactive area according to the manufacturer's instructions. The test strip was not allowed to contact the tooth and its entry into the sulcus was also avoided. After measuring the GCB, the CFB was immediately measured using the same glucometer. The pad of the finger was wiped with alcohol, allowed to dry and then punctured with a sterile lancet. A CFB sample was collected on the test strip. The GCB and CFB glucose readings were recorded.

## Results and Discussion:

A descriptive statistical analysis has been carried out in the present study. Significance was assessed at a 5% level of significance. Among the 200 patients, 70% comprised of periodontitis and 30%, gingivitis. Out of the 200 tested patients, 24 patients revealed elevated blood glucose levels. The glucose measurement from the GCB sample ranges from 60 to 234 mg/dL with a mean of 97.18±60.13 mg/dL. Glucose for CFB samples ranges from 87 to 289 mg/dL with a mean of 133.9±61.1mg/dL (Table 1). Pearson's correlation coefficient showed a positive correlation between GCB and CFB (Table 2). 'r' value of 0.93 shows a very strong correlation between CFB and GCB results, which is highly statistically significant (P<0.0001). Diabetes mellitus are classified into type I-Insulin dependent diabetes mellitus (IDDM) and type II- Non-insulin-dependent diabetes mellitus (NIDDM) [10]. Diabetes in 2030, is expected to affect 578 million people and 700 million people will be affected till 2045 [11]. By 2030, India, China and the United States will have the highest prevalence of diabetes [11]. It is proven that the prevalence of the DM is greater among individuals with periodontitis than healthy individuals. Gingival crevicular blood oozes during the routine oral examination can be used as an alternative for blood glucose-level estimation because studies have shown that, when compared with the usual method of collecting blood, gingival crevicular blood is a painless procedure and can be used for early diagnosis of diabetes [12]. So the aim of the present study is to use this extravagated blood from the gingival crevice for the early detection of DM with the help of self-monitoring device (Glucometer). To validate this technique we compare results with the blood glucose level obtained from capillary finger blood using same glucometer. The results of the present study revealed a strong correlation between GCB and CFB findings which is in accordance with the study conducted by Gaikwad *et al.* [13] Bhavsar *et al.* [14] compared gingival crevicular blood and venous blood and found no significant results, whereas in our study comparing finger prick blood and gingival crevicular blood, the results are statistically significant (p<0.0001), which was in agreement with the study by Kaur *et al.* [15] and Rapone *et al.* [16]. Sarlati *et al.* [17] reported that gingival crevicular blood is useful for testing blood glucose during routine periodontal examination in subjects with DM and periodontitis. Khader *et al.* [18] reported that GCB can be an acceptable source for measuring the blood glucose level.

## Conclusion:

Gingival crevicular blood obtained during a routine oral examination can be a used as a potential source for screening of blood glucose using a glucometer in population with an unknown history of DM.

## Limitations of the study:

Further studies with a larger sample size are recommended to conclude the importance of gingival crevicular blood.

## Financial support and sponsorship:

Nil.

## Figures and Tables

**Table 1:** Descriptive statistics

**Variables**	**Range**	**Mean**
GCB	60 - 234 mg/dL	97.18±60.13 mg/dL
CFB	87 to 289 mg/dL	133.9±61.1 mg/dL

**Table 2:** P Value

**Glucose level**	**P Value**
GCB vs CFB	0.93 <0.0001
